# Supplementary material for: Prospects for silvicultural enhancement of fire resistance in mesic westside forests of the Pacific Northwest
Source: PLoS One. 2025 Sep 8;20(9):e0332158. doi: 10.1371/journal.pone.0332158 (PMC12416676; doi:10.1371/journal.pone.0332158)
Supplement: S1 Appendix — (DOCX) [file pone.0332158.s001.docx]

Prospects for silvicultural enhancement of fire resistance in mesic westside forests of the Pacific Northwest.

Sebastian U. Busby and Jeremy S. Fried

S1 Appendix:

# Forest inventory data

We used data from a statistically representative and spatially balanced sample of field-measured Forest Inventory and Analysis (FIA) plots, from the most recently available evaluation identifier (with panels assessed in 2008-2019), as inputs to forest growth and yield model simulations in the Forest Vegetation Simulator (FVS). To identify and obtain a list of FIA plots within our study area boundaries, we used ArcGIS [1] to conduct a spatial overlay (intersection) of the public FIA plot coordinates and a polygon shapefile of the FVS Western Cascades (WC) and Pacific Coast Range (PN) variant boundaries. From this selection, we excluded plots that intersected the Willamette Valley and Klamath Mountains ecoregion boundaries, as defined by the Environmental Protection Agency (EPA) Level 3 Ecoregion boundaries, given our study’s focus on mesic montane conifer forest types. We also excluded plots located within urban growth boundaries of the Seattle Washington metropolitan area or on islands (i.e., San Juan islands) with no roadway connection (i.e., a bridge) to wood processing facilities. See Fig 1 for a map of the FIA plots (fuzzed locations) modeled in this study, symbolized by owner group.

Sampled area within each FIA plot is partitioned into separate “conditions” when delineation-qualifying differences (e.g., in owner group, forested status, reserve status, forest type, stand size class, tree density class, topographic features) exist and minimum condition size and shape criteria are satisfied. Site attributes such as slope, site quality, stand age, forest type and landowner group are defined and collected at the condition level, and each condition can be thought of as a forest “stand” that represents a statistically based sample corresponding to a definable number of forested acres within a broader landscape. We used FIA conditions, hereafter referred to as stands, as the basic simulation and analysis unit in this study.

We used the Bioregional Inventory Originated Simulation Under Management (BioSum) analysis framework and workflow management software [2] to prepare and pre-process FIA stand data for simulation in FVS and to generate and analyze economic data associated with FVS simulation output. Applying BioSum to Oregon and Washington state-level SQLite-format FIA databases (FIADB), we generated SQLite FVS input files containing the standard FVS_STANDINIT_COND (i.e., stand-scale attributes) and FVS_TREEINIT_COND (i.e., tree-level attributes) data tables, starting with the versions of these tables provided in FIADB. We excluded FIA conditions that were non-forest (<10% canopy cover) or had a sampled condition proportion < 0.25; such conditions often contain too few trees to effectively represent actual forest composition and structure. The area represented by the conditions excluded by the condition proportion threshold was reallocated proportionally to the remaining conditions, resulting in a sample consisting of 6396 forested conditions (stands). We included trees in the seedling table to account for potential ladder-fuel development over time, and previous tree diameters and heights when working with remeasured inventory plots to support FVS self-calibration of its tree growth models toward localized empirical growth patterns. To improve model estimation of tree growth and mortality, we ensured that each FIA stand record had a valid potential vegetation habitat type code in the FVS input file (FVS_STANDINIT_COND.PV_CODE field). When the PV_CODE field was null or contained a code regarded as invalid for FVS, we used stand inventory conditions (species composition, location, and site productivity) to crosswalk each stand to an appropriately matched code. We also calculated and populated a maximum stand density index (MaxSDI) value for each stand in the FVS input file (FVS_STANDINIT_COND.MAXSDI field), following methods and equations outlined by [3] for Douglas-fir or western hemlock dominated westside Pacific Northwest forests, utilizing the non-fuzzed (i.e., actual) FIA plot coordinates.

# Silvicultural treatments

To represent and simulate business-as-usual (BAU) forest management practices in westside forests, we constructed silvicultural prescriptions (treatments; Rx) based on data obtained from survey and interview responses of regional large forest landowners (stratified random sample of Private Corporate, Federal, State, and Tribal groups), to support the USDA Forest Service Pacific Northwest Research Station’s Westside Fire Research Initiative. Survey and interview questions asked specifically about common agency-level forest management practices (e.g., harvest systems, rotation lengths, commercial and non-commercial thinning parameters, species retention and planting preferences, thinning and harvest triggers and targets). Formal ethics approval for this study was not obtained because all researchers facilitating surveys and interviews were U.S. Forest Service scientists. The Forest Service has no ethics approval or Institutional Review Board (IRB) process but does have a scientific integrity policy that was followed.

Interviews with research participants were conducted virtually, began in April 2021, and included pilot testing before the interview guide that was finalized in June of the same year; interviews continued throughout 2022. Participants provided informed consent in two ways. Study participants were recruited via email, and occasionally with a follow-up phone call if there was no email response after several attempts. In the recruitment email, the study purpose was described and the topics planned to be covered in the interview. It was clear that participation was voluntary. It was stated that the information they provided in the interview would be kept confidential (it was combined with responses from other study participants for reporting purposes). They gave their consent to participate in the study (or not) via email. Potential study participants were also invited to ask any questions or express any concerns prior to the interview. Before starting the interview, an overview of the study and its purpose was provided, and how the interview data would be used, and confidentiality policy restated. Consent to record the interview was obtained and participants provided verbal consent at this time. If they were not comfortable having the interview recorded, we did not do so. We developed a confidentiality policy document for interviewees at the request of one sub-group (Private Corporate forest landowners) and shared it with them to facilitate the informed consent process. Individual survey and interview responses were generalized to and analyzed at the ownership level.

Below we describe the process for transforming survey data and interview transcripts into a set of silvicultural prescriptions representing BAU forest management. First, we partitioned surveys by ownership and ecoregion and reviewed each for completeness and usability of responses (i.e., does it contain actionable information?). Where survey responses were left unanswered or unclear, we reviewed interview transcripts and interpreted interview responses to replace null or unclear survey responses, when possible. Next, we identified silvicultural parameters, per ownership and ecoregion group, most frequently mentioned (i.e., modal) across surveys and interviews and relied on them to craft silvicultural prescriptions. Where silvicultural parameters provided by interviewees were evenly distributed within a narrow range, we used the midpoint of the value range to inform prescriptions. Where responses indicated a broader range, we developed multiple prescriptions using the ends and midpoint of value ranges. Where harvest triggers and residual density targets varied in the stand density metrics described (e.g., trees per acre, basal area, relative density index, stand density index), we used a single relativized density metric across prescriptions to ensure tractable analysis: percent of maximum stand density index (% of MaxSDI). Stand MaxSDI varies according to species composition and site productivity, accounting for variation among stands in the level of stem density they can support (e.g., without incurring competition-induced tree mortality). Given the diversity of forest types and productivity gradients present in our study area, using this relativized metric significantly reduced the number of prescriptions that would need to be crafted and analyzed.

BAU Treatments were associated with representative examples of what can be loosely described as even- or uneven-aged management styles, differentiated by the former ultimately involving a stand-replacing harvest (e.g., clearcut and replant) and the latter a sequence of individual tree selection harvests, with trees thinned evenly across diameter classes at each entry, to maintain recruitment of new, younger growing stock over time. To contrast BAU management and treatments, which largely focus on maximizing stand growth or enhancing old growth structural characteristics, we developed a suite of “fire-aware” even- and uneven-aged style treatments aimed at enhancing stand-scale fire resistance. Fire-aware treatments are designed to enhance fire resistance over BAU by (1) lowering density triggers for commercial thinning entries and thinning to lower residual densities, (2) thinning from below and replanting at lower densities for even-aged style treatments, and (3) reducing surface fuel loading and ladder fuels by thinning ladder fuels (non-merchantable trees < 15cm diameter) and removing residues, pile burning residues, or broadcast burning.

Finally, to ensure our BAU and fire-aware silvicultural prescriptions were realistic, we elicited feedback from regional silviculturists and revised, removed, or added prescriptions as recommended. Once finalized, each silvicultural prescription was coded as an FVS keyword control parameters file, to control simulation in FVS. We tested each prescription file in FVS on a subset of stands and reviewed the simulation outputs to verify that silvicultural activities were being applied as intended, as a quality assurance step in advance of running simulations of the whole landscape. See Table 1 for the full list of silvicultural prescriptions developed and simulated in this study.

# References

1. ESRI. ArcGIS Pro. Version 3.4.1. Environmental Systems Research Institute, Redlands CA. 2024.
2. Fried JS, Potts LD, Loreno SM, Christensen GA, Barbour RJ. Inventory-Based Landscape-Scale Simulation of Management Effectiveness and Economic Feasibility with BioSum. Journal of Forestry. 2017 Jul 31; 115(4): 249–57.
3. Heiderman RR, Kimsey MJ. A species-specific, site-sensitive maximum stand density index model for Pacific Northwest conifer forests. Can J For Res. 2021 Aug; 51(8): 1166–77.
