# Supplementary figures and images for: Prospects for silvicultural enhancement of fire resistance in mesic westside forests of the Pacific Northwest
Source: PLoS One. 2025 Sep 8;20(9):e0332158. doi: 10.1371/journal.pone.0332158 (PMC12416676; doi:10.1371/journal.pone.0332158)

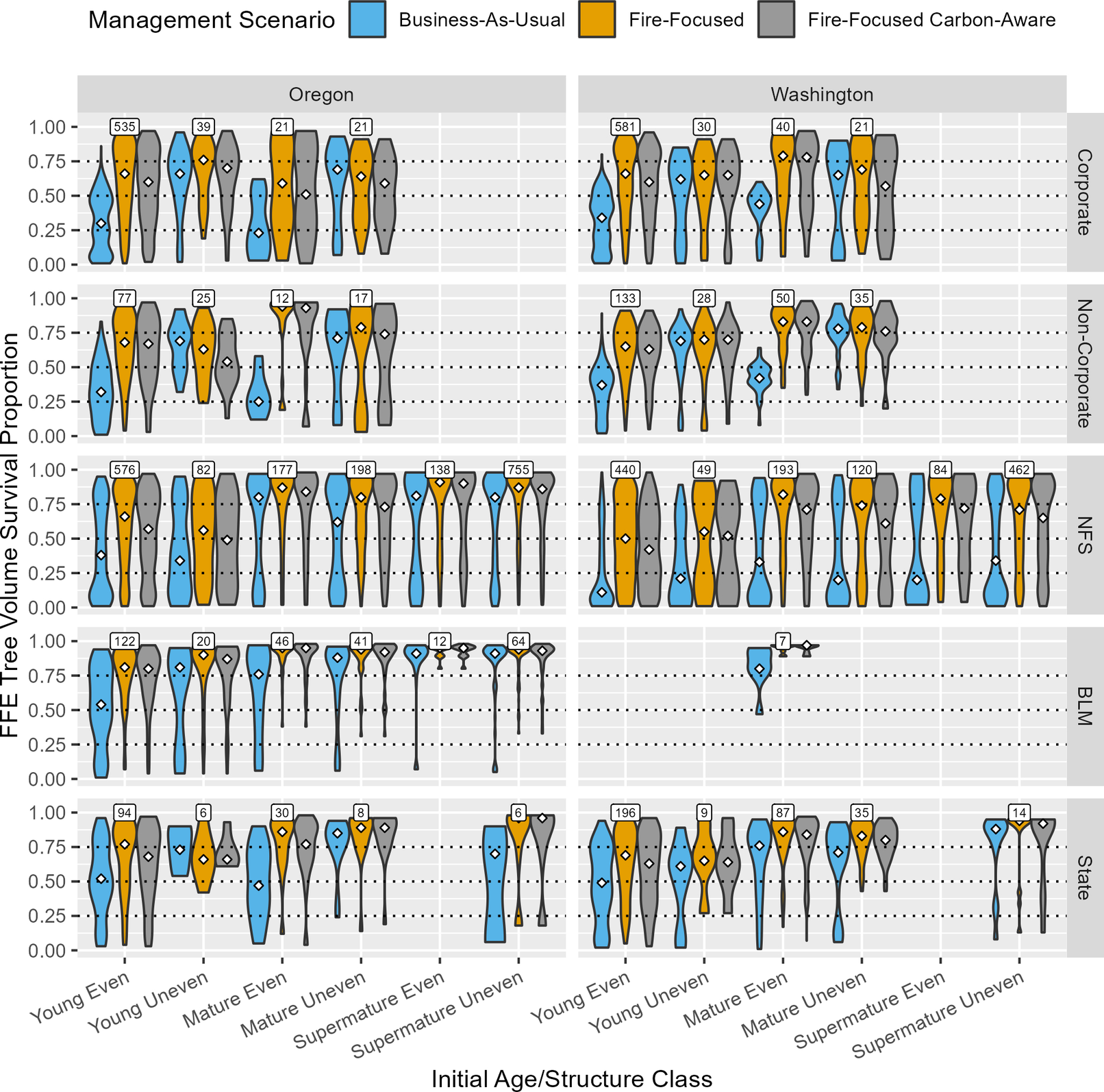

Supplement: S1 Fig — White diamonds indicate the area-weighted median value across stands in each category. Boxed values posted above violin clusters show the sample size (number of stands) associated with each stratum; stratum with 5 or fewer sample stands are not included in this chart. Table 2 describes the response variable and its calculation. (TIF) [file pone.0332158.s002.tif]

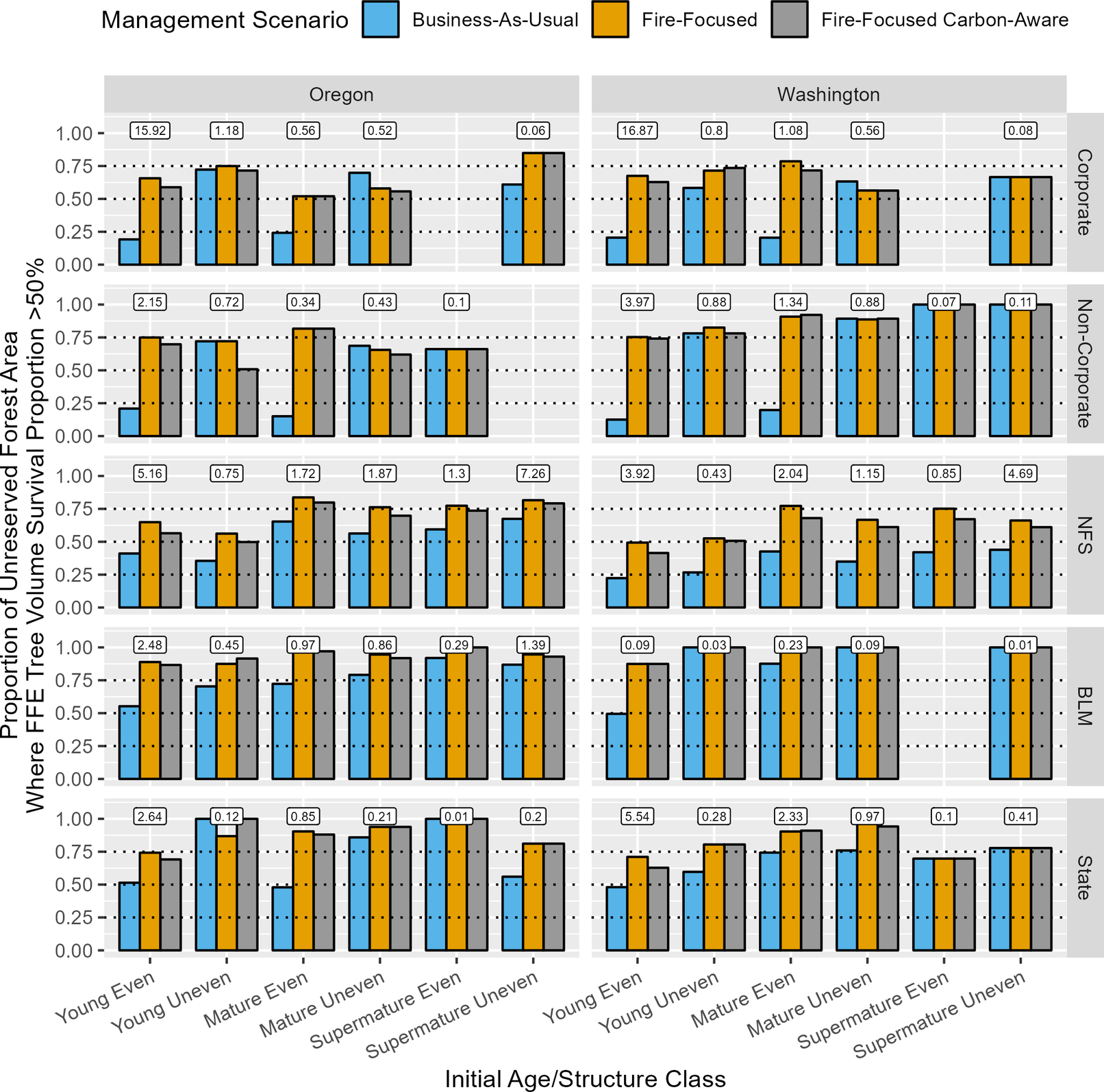

Supplement: S2 Fig — Boxed values posted above bar cluster strata represent the percent of total unreserved forest area associated with each stratum. Table 2 describes the response variable and its calculation. (TIF) [file pone.0332158.s003.tif]

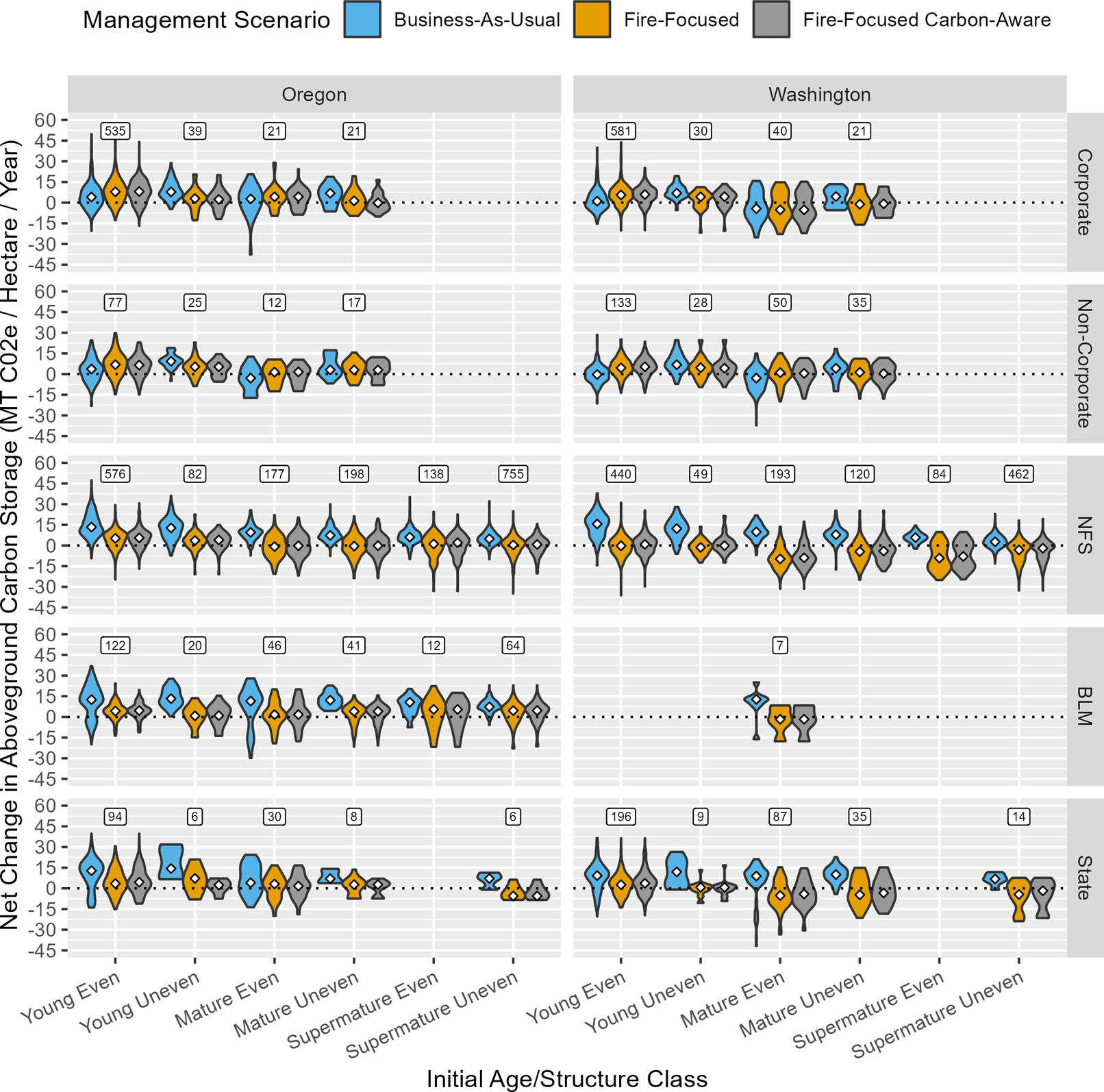

Supplement: S3 Fig — White diamonds indicate the area-weighted median value across stands in each category. Boxed values posted above violin clusters show the sample size (number of stands) associated with each stratum; stratum with 5 or fewer sample stands are not included in this chart. Table 2 describes the response variable and its calculation. (TIF) [file pone.0332158.s004.tif]

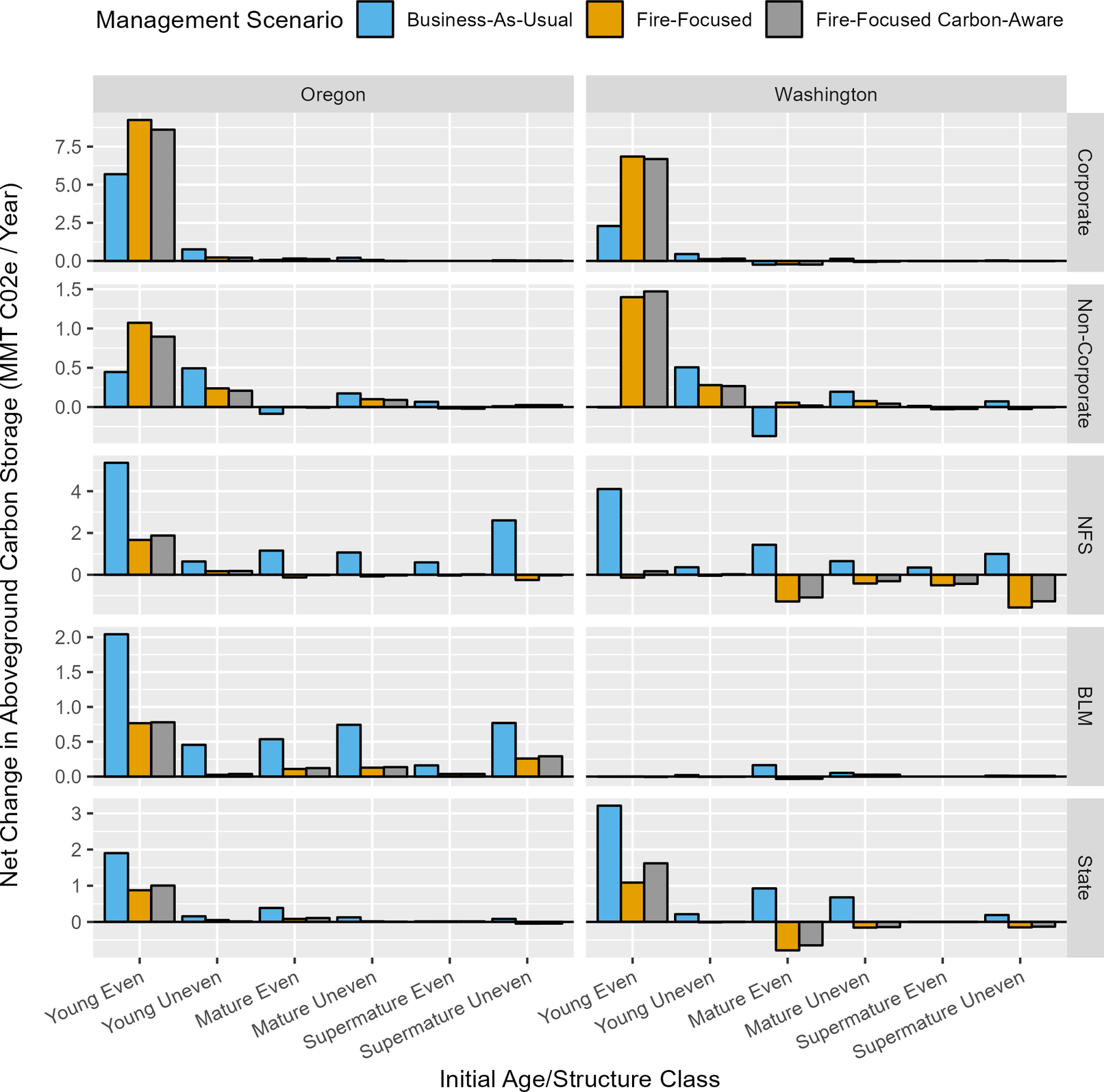

Supplement: S4 Fig — Note that Y-axis scales differ by ownership. Table 2 describes the response variable and its calculation. (TIF) [file pone.0332158.s005.tif]

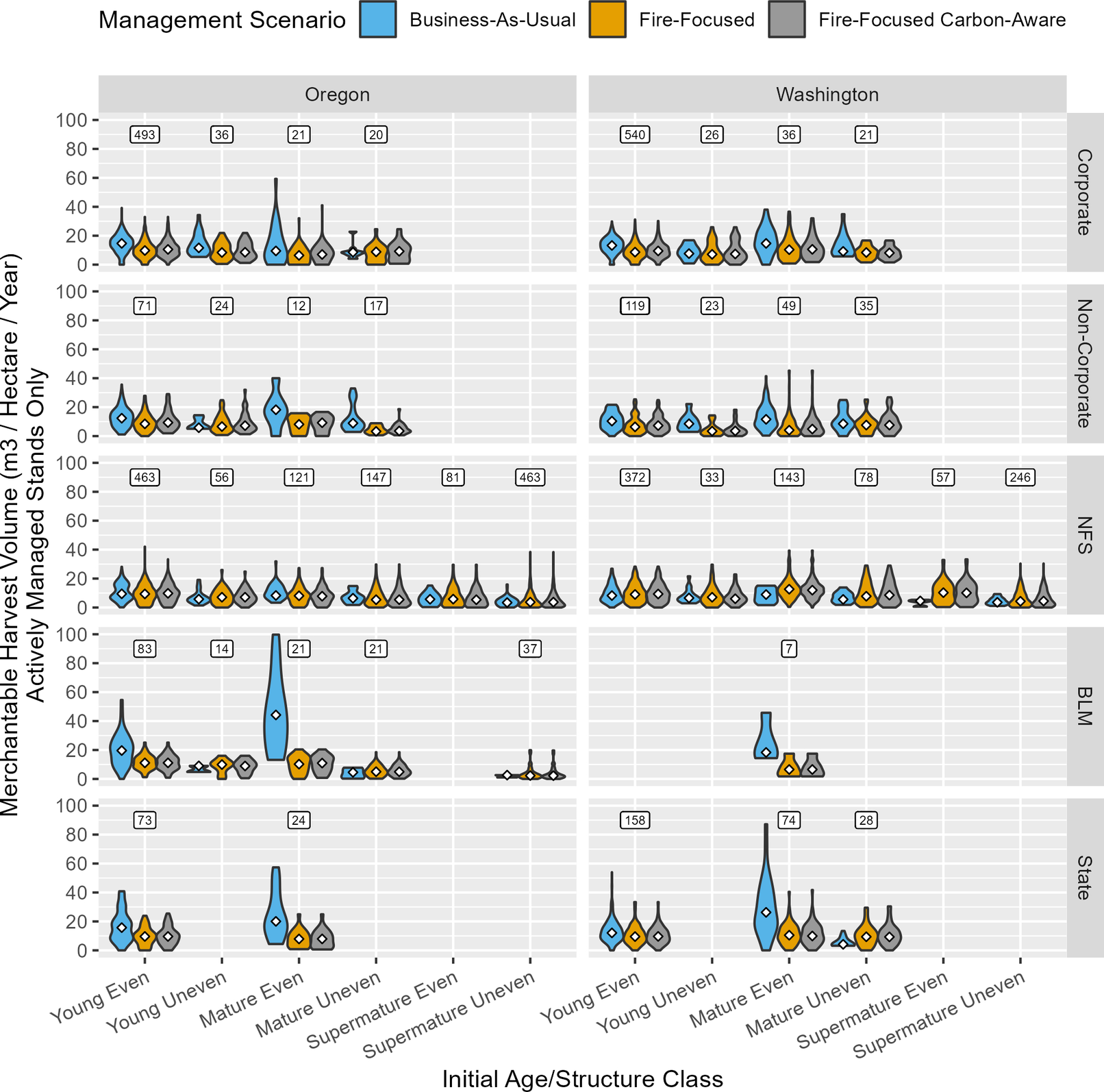

Supplement: S5 Fig — White diamonds indicate the area-weighted median value across stands in each category. Boxed values posted above violin clusters show the sample size (number of actively managed stands across management scenarios) associated with each stratum; stratum with 5 or fewer sample stands are not included in this chart. Table 2 describes the response variable and its calculation. (TIF) [file pone.0332158.s006.tif]

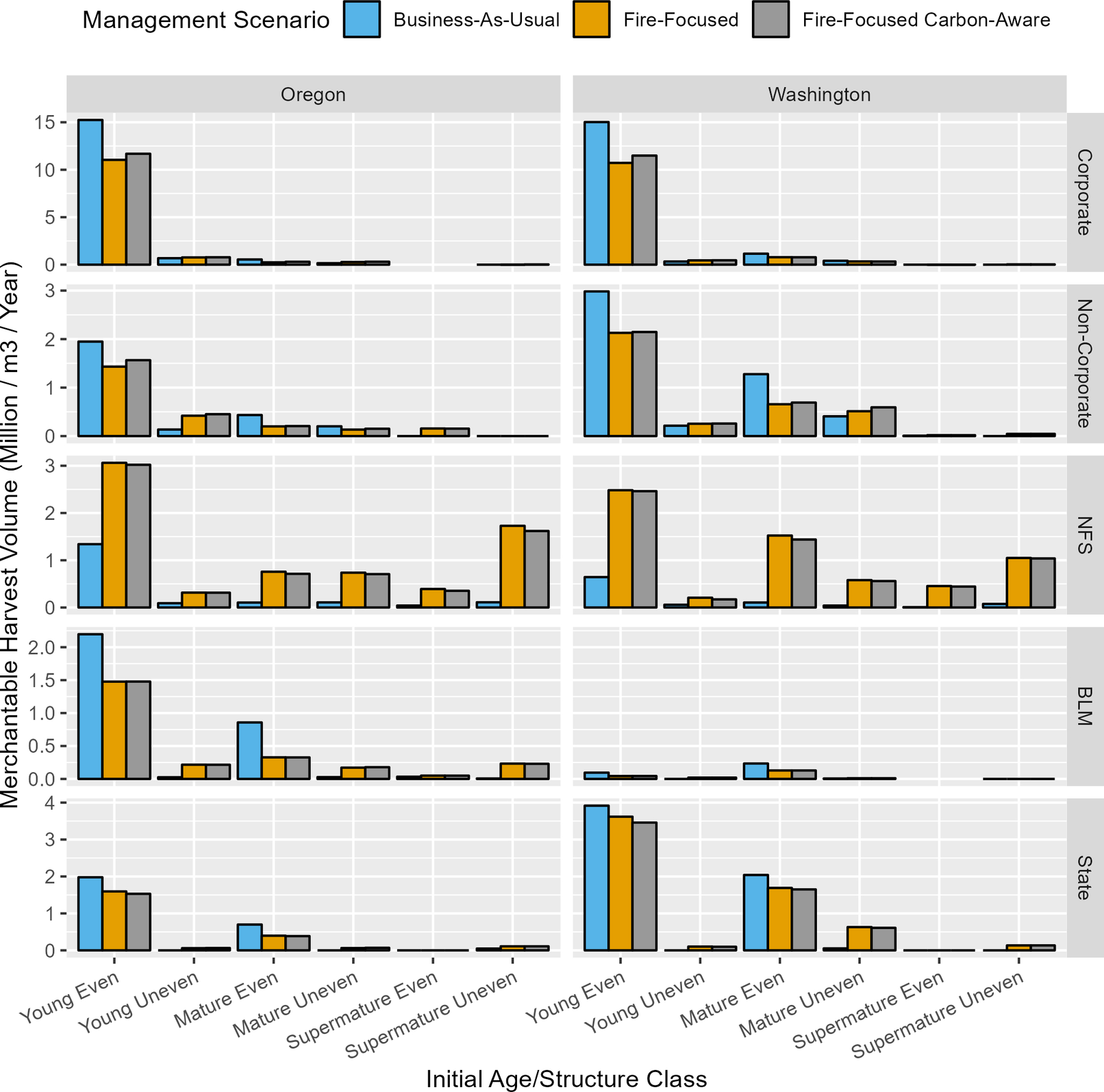

Supplement: S6 Fig — Note that Y-axis scales differ by ownership. Table 2 describes the response variable and its calculation. (TIF) [file pone.0332158.s007.tif]

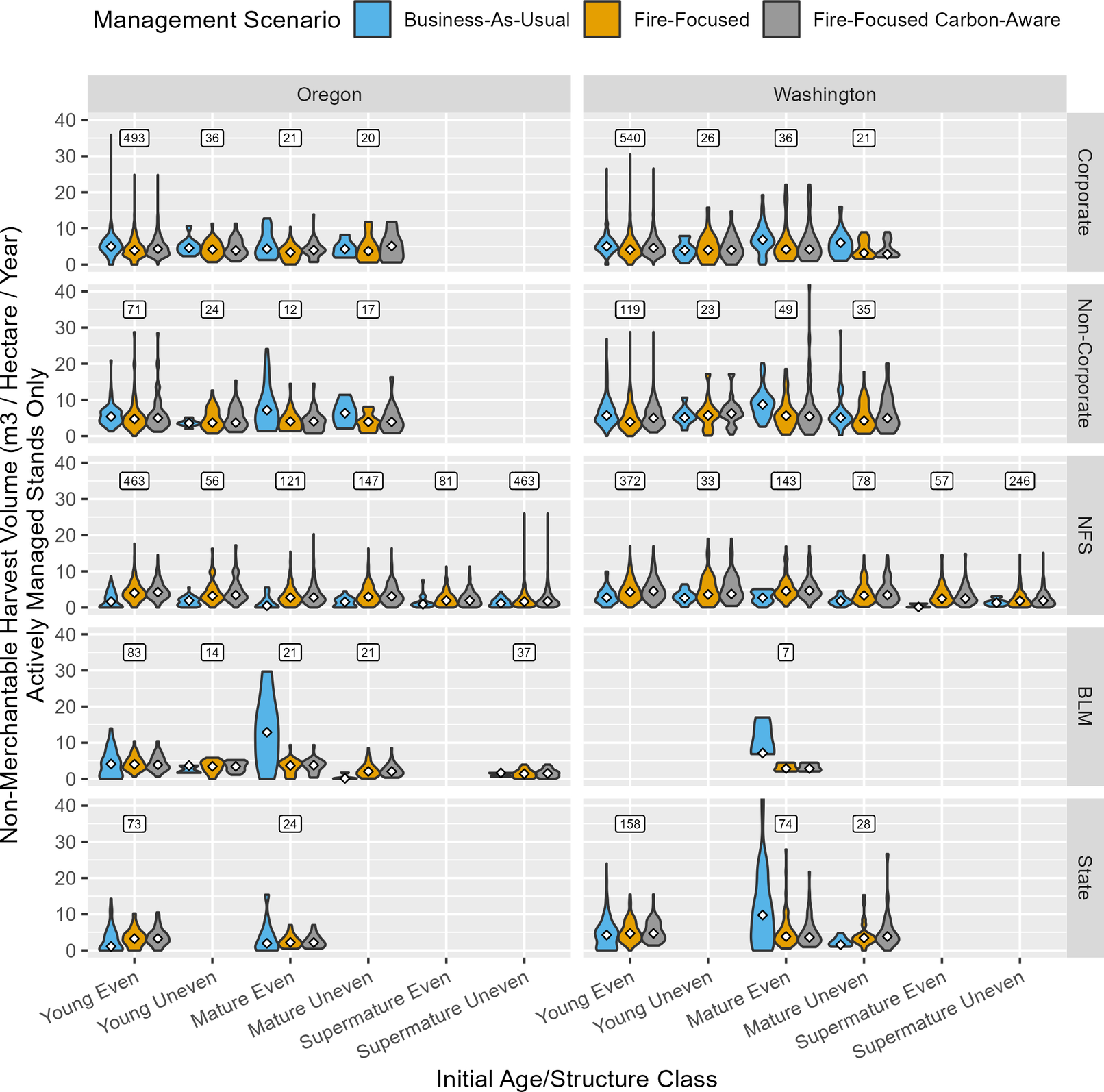

Supplement: S7 Fig — White dots indicate the area-weighted median value across stands in each category. Boxed values posted above violin clusters show the sample size (number of actively managed stands across management scenarios) associated with each stratum; stratum with 5 or fewer sample stands are not included in this chart. Table 2 describes the response variable and its calculation. (TIF) [file pone.0332158.s008.tif]

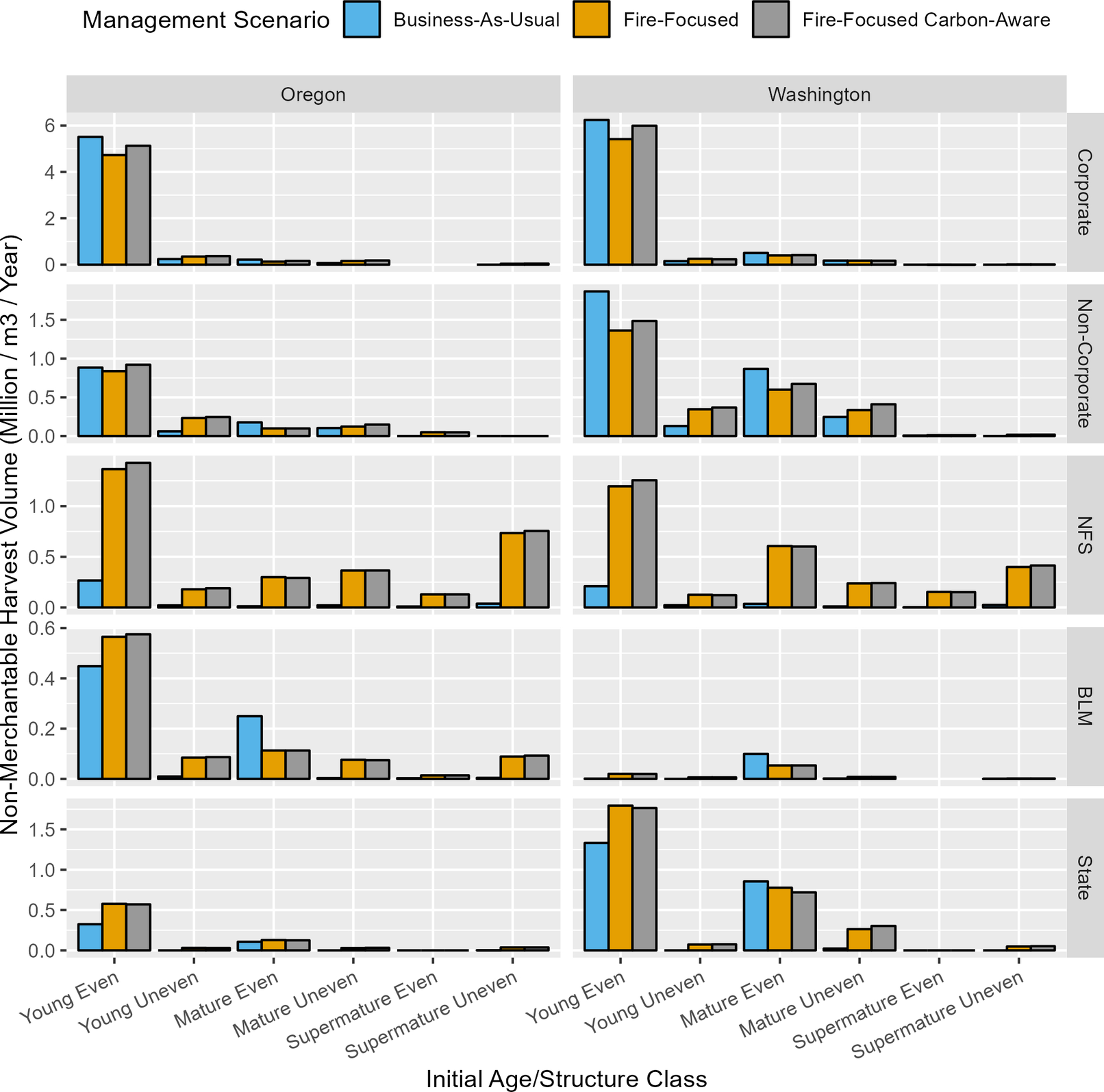

Supplement: S8 Fig — Note that Y-axis scales differ by ownership. Table 2 describes the response variable and its calculation. (TIF) [file pone.0332158.s009.tif]
