# Supplementary material for: Prospects for silvicultural enhancement of fire resistance in mesic westside forests of the Pacific Northwest
Source: PLoS One. 2025 Sep 8;20(9):e0332158. doi: 10.1371/journal.pone.0332158 (PMC12416676; doi:10.1371/journal.pone.0332158)
Supplement: S1 Table — Costs were sourced from regional foresters and fuel treatment experts. (DOCX) [file pone.0332158.s010.docx]

Prospects for silvicultural enhancement of fire resistance in mesic westside forests of the Pacific Northwest.

Sebastian U. Busby and Jeremy S. Fried

**S1 Table.** Additional harvest costs, by treatment type, used to parameterize the Processor module of BioSum. Costs were sourced from regional foresters and fuel treatment experts.

| Treatment | Cost (2022 USD / hectare) |
| --- | --- |
| Pileburn at landing | 247 |
| Pileburn in unit | 618 |
| Broadcast burn | 371 |
| Site prep and replant post-clearcut | 988 |
