# Supplementary material for: Prospects for silvicultural enhancement of fire resistance in mesic westside forests of the Pacific Northwest
Source: PLoS One. 2025 Sep 8;20(9):e0332158. doi: 10.1371/journal.pone.0332158 (PMC12416676; doi:10.1371/journal.pone.0332158)
Supplement: S2 Table — Values were estimated by a forest economist (Mike Buffo, Mason, Bruce and Girard) using the MBGTools model. (DOCX) [file pone.0332158.s011.docx]

Prospects for silvicultural enhancement of fire resistance in mesic westside forests of the Pacific Northwest.

Sebastian U. Busby and Jeremy S. Fried

**S2 Table.** Delivered timber values by tree diameter range and species group, averaged across subregions, used to parameterize the Processor module of BioSum. Values were estimated by a forest economist (Mike Buffo, Mason, Bruce & Girard) using the MBGTools model.

|  | Grade weighted log value (2022 USD / m3) by species group | | | | | |
| --- | --- | --- | --- | --- | --- | --- |
| Tree diameter range (inches diameter at breast height) | Douglas-fir | Hemlock/Spruce/Fir | Cedar | Pine | Red Alder | Other Hardwoods |
| 0 - 6 | 0.00* | 0.00* | 0.00* | 0.00* | 0.00* | 0.00* |
| 6.1-12 | 88.64 | 67.10 | 143.03 | 45.20 | 69.57 | 0.00* |
| 12.1-20 | 104.53 | 88.29 | 165.63 | 49.44 | 73.10 | 0.00* |
| 20.1-30 | 139.14 | 104.89 | 189.29 | 55.44 | 73.10 | 0.00* |
| >30.1 | 162.10 | 119.72 | 241.55 | 66.39 | 73.10 | 0.00* |
| *Logs are allocated to chips | | | | | | |
