# Supplementary material for: Prospects for silvicultural enhancement of fire resistance in mesic westside forests of the Pacific Northwest
Source: PLoS One. 2025 Sep 8;20(9):e0332158. doi: 10.1371/journal.pone.0332158 (PMC12416676; doi:10.1371/journal.pone.0332158)
Supplement: S3 Table — Fire effects were modeled in the Forest Vegetation Simulator (FVS) using the Fire and Fuels Extension (FFE). (DOCX) [file pone.0332158.s012.docx]

Prospects for silvicultural enhancement of fire resistance in mesic westside forests of the Pacific Northwest.

Sebastian U. Busby and Jeremy S. Fried

**S3 Table.** Wildfire simulation weights used to calculate weighted average values at each simulation timestep, across simulation output metrics, to represent a percent area burned per decade fire prevalence scenario. Fire effects were modeled in the Forest Vegetation Simulator (FVS) using the Fire and Fuels Extension (FFE).

|  | Wildfire Simulation Weights | | | | |
| --- | --- | --- | --- | --- | --- |
| Fire Prevalence Scenario | Mild-Moderate Weather^1^ Wildfire Year 11 | Severe Weather^2^ Wildfire Year 11 | Mild-Moderate Weather^1^ Wildfire Year 31 | Severe Weather^2^ Wildfire Year 31 | No Wildfire |
| 5% Area Burned per Decade | 0.02 | 0.08 | 0.02 | 0.08 | 0.80 |
| ^1^Assumes “moist” fuel moisture content, average temperature of 21°C, and average wind speed of 2.2 m/s. | | | | | |
| ^2^Assumes “very dry” fuel moisture content, average temperature of 21°C, and average wind speed of 6.7 m/s. | | | | | |
