# Supplementary material for: Prospects for silvicultural enhancement of fire resistance in mesic westside forests of the Pacific Northwest
Source: PLoS One. 2025 Sep 8;20(9):e0332158. doi: 10.1371/journal.pone.0332158 (PMC12416676; doi:10.1371/journal.pone.0332158)
Supplement: S4 Table — (DOCX) [file pone.0332158.s013.docx]

Prospects for silvicultural enhancement of fire resistance in mesic westside forests of the Pacific Northwest.

Sebastian U. Busby and Jeremy S. Fried

**S4 Table.** Silvicultural treatment selection optimization logic by ownership and initial age/structure class associated with the business-as-usual (BAU) and fire-focused (FF) forest management scenarios.

| **Business-As-Usual (BAU) Forest Management Scenario Treatment Selection Logic** | | | | | | |
| --- | --- | --- | --- | --- | --- | --- |
|  | **Initial Age/Structure Class** | | | | | |
| **Ownership** | Even-Age Young | Even-Age Mature | Even-Age Supermature | Uneven-Age Young | Uneven-Age Mature | Uneven-Age Supermature |
| NFS | *OGSI* | *OGSI* | *OGSI* | *OGSI* | *OGSI* | *OGSI* |
| BLM | *NPV* | *NPV* | *OGSI* | *OGSI* | *OGSI* | *OGSI* |
| State | *NPV* | *NPV* | *OGSI* | *OGSI* | *OGSI* | *OGSI* |
| Private | *NPV* | *NPV* | *OGSI* | *NPV* | *NPV* | *OGSI* |
| **Fire-Focused (FF) Forest Management Scenario Treatment Selection Logic** | | | | | | |
|  | **Initial Age/Structure Class** | | | | | |
| **Ownership** | Even-Age Young | Even-Age Mature | Even-Age Supermature | Uneven-Age Young | Uneven-Age Mature | Uneven-Age Supermature |
| NFS | *Resist* | *Resist* | *Resist* | *Resist* | *Resist* | *Resist* |
| BLM | *Resist* | *Resist* | *Resist* | *Resist* | *Resist* | *Resist* |
| State | *Resist* | *Resist* | *Resist* | *Resist* | *Resist* | *Resist* |
| Private | *ResistNoGO* | *ResistNoGO* | *Resist* | *ResistNoGO* | *ResistNoGO* | *Resist* |
| *Young: age < 60yrs; Mature: age 60-120yrs; Supermature: age > 120yrs*  *OGSI*: Select treatment (including grow-only) that maximizes 40-year weighted average Old Growth Structure Index (OGSI) value. | | | | | | |
| *NPV*: Select treatment (excluding grow-only) that maximizes 40-year net present value (sum) excluding grow-only (GO). | | | | | | |
| *Resist:* Select treatment (including grow-only) that maximizes 40-year weighted average FOFEM-predicted tree volume survival proportion to wildfire. | | | | | | |
| *ResistNoGO*: Select treatment (excluding grow-only) that maximizes 40-year weighted average FOFEM-predicted tree volume survival proportion to wildfire.  Note: Logic for selections under the fire-focused carbon-aware (FFCA) scenario is identical to that for Fire-focused (FF). | | | | | | |
