# Supplementary material for: Prospects for silvicultural enhancement of fire resistance in mesic westside forests of the Pacific Northwest
Source: PLoS One. 2025 Sep 8;20(9):e0332158. doi: 10.1371/journal.pone.0332158 (PMC12416676; doi:10.1371/journal.pone.0332158)
Supplement: Attachment — Submitted filename: busby-fried_review1_2025.docx [file pone.0332158.s014.docx]

**Summary:**

In this manuscript, Busby and Fried seek to characterize potential tradeoffs between fire resistance, carbon, and economic goals with regard to forest management strategies in the western Cascades, using a simulation modeling approach. The authors compare business-as-usual and fire-aware management approaches across a variety of land ownership and forest structure scenarios. The authors note potential considerations for future management based on these results.

I think that this study will be of interest to both researcher and manager readership of *PLOS ONE*, and is appropriate for this journal’s aims and scope. Overall I think this study is timely and well-designed, but would benefit from additional edits for clarity and conciseness before publication. I have outlined my recommendations below and look forward to seeing the next version of the manuscript.

**Main comments (essential revisions):**

1. At 26 pages, 15 figures, and 5 tables, the paper is quite long and in places feels unfocused. I can appreciate the challenge with concisely reporting findings for such a large, multidimensional model. That said, I suggest editing with an eye toward trimming the paper back to its main story. I have made some specific suggestions below, but consider limiting figures to one per results section (i.e., one carbon figure, one fire resistance figure, one economic figure), paneling or summarizing results as necessary. I would also consider actually adding a figure to the tradeoffs section (either in addition to or in place of the table). To me this is the “star” of the paper and it would be great to have a visual here that is digestible at a glance.
2. The First Order Fire Effects Model is a major part of this study. However, as far as I can tell the first mention of this model is in Table 3, with no information on how this model was used or parameterized in the methods. I feel that I cannot adequately evaluate this portion of the study without more information.
3. It is not clear to me whether each individual model scenario was run multiple times or just once. If multiple times, how much variability in outcomes is there per scenario? If just once, how did you account for stochastic effects in the model?
4. Although results are thoroughly described, the paper is lacking in statistical testing in general. It would be especially helpful to see some additional stats in the tradeoffs section of the results. Are outcomes significantly different by initial forest structure and/or management scenario?

**Minor comments (desirable revisions):**

1. The simulation period (40 years) does not include the full range of a single rotation period (35-60 years), which I feel is a crucial caveat to interpreting the results. While this is addressed in Lines 738-750 of the discussion, I would like to see a bit more discussion of the effects of simulation length on outcomes other than carbon alone (i.e., potential differences between short-term and long-term tradeoffs, especially in the context of repeat burns). Although this is touched on in the discussion, it may also be worth briefly noting in the methods (Line 295) why 40 years was chosen.
2. There are several sentences in the methods and results section that are difficult to parse – I have outlined a few in the following section. Consider breaking up clauses into sentences and reducing parentheses use where possible.
3. There are a great many acronyms in this paper. Although they make sense in cases where they are defined appropriately and used frequently (like BAU, FF, and FFCA), others (like LCA and FFE) are only used a handful of times and should be written out.
4. In general I would like to see more quantitative reporting in the results, especially if the number of figures is reduced. Statements such as “exceptionally low” (Line 490), “modest improvements” (Line 511), “lower tree survival” (Line 515), “more modest” (Line 535), etc. should be replaced with the corresponding value or statistic where feasible.
5. It was difficult for me to parse that the FF and FACC scenarios fall under the FA category presented in Table 1. I would recommend clarifying this in 397-399.

Specific text comments

- Lines 40-51: This paragraph lacks citations; I would recommend at least adding an appropriate citation for the sentence beginning with “Westside forests…” (lines 44-47).
- Lines 107-109: Not an edit – just noting that I really like this sentence.
- Lines 144-169: Some of this information is already stated in the introduction; I recommend condensing where possible.
- Lines 213-215: I am not sure what is meant by “used stand inventory conditions to select and enter an appropriate code”.
- Lines 220-247: This paragraph is very long – consider breaking up at line 231 (“Interviews with…”)
- Lines 251-254: I am not sure what is meant by this sentence.
- Line 300: Clarify why these wind speeds were selected.
- Lines 320-328: Consider moving these lines to the “Wildfire Activity and Prevalence” section.
- Lines 386: Why was Year 41 unevenly weighted?
- Line 475: Consider changing 18% unmanaged to 82% managed, for consistency and easier comparison with the preceding paragraphs.
- Line 550: Should this be FF instead of FA?
- Line 578 and elsewhere: Consider changing “stand level” and “landscape level” to “stand scale” and “landscape scale” for clarity.
- Lines 635-650: Lots of background information here before a reference to your findings – consider removing this paragraph altogether or moving to the introduction.

Figures & tables

- Figure 1 – the color schemes here are a bit confusing, as the colors for “State” and “Mature Even”, “Private” and “Young Uneven”, and “NFS” and “Supermature Even” are very similar. It is easy for readers to look at the wrong legend.
- As previously noted, consider further paneling violin plots per result topic or moving some of these figures to the supplement.
- Table 1 – define what “trigger” means in the model context either in the table caption or somewhere in the text.
- Table 4 could also be moved to the supplement as it is not strictly necessary for interpreting the modeling process, in my opinion.
