# Supplementary material for: Prospects for silvicultural enhancement of fire resistance in mesic westside forests of the Pacific Northwest
Source: PLoS One. 2025 Sep 8;20(9):e0332158. doi: 10.1371/journal.pone.0332158 (PMC12416676; doi:10.1371/journal.pone.0332158)
Supplement: Attachment — Submitted filename: Response to Reviewers.docx [file pone.0332158.s016.docx]

**Author responses to reviewer comments for Busby & Fried 2025 – PLOS One**

**All author responses are noted in blue colored text below**

**Reviewer #1**

Reviewer #1: This manuscript addresses a significant knowledge gap related to the impacts of fuel treatments or “fire aware” management in moist, westside forests of the PNW. As such, it has significant value to land management planning in the region. The authors modeling approach appears to be appropriate, although there is perhaps room for added analysis of results that are presented as stand-scale averages.

Thank you for the comment. All results in this study include stand-scale outcomes summarized via value distribution (violin) plots that include area-weighted median values via figure graphics (Figures 4, 7, 9, S1, S3, S5, S7), and stand-scale tradeoffs between outcome variables are also reported as area-weighted medians in Table 5 (now Table 3). The median is typically a more appropriate summary statistic to report than the mean (average), when response distributions are non-normal, which is common across the response (outcome) variables analyzed in this study.

Throughout: this is a minor point, but when referring to categories of forest management or silvicultural treatments, the commonly-used terminology is “even-aged” and “uneven-aged” rather than “even-age” and uneven-age”. See for instance, Nyland, RD. 2016. Silviculture: concepts and applications. 3rd Ed. Waveland or Deal, R. 2017. Dictionary of Forestry. Society of American Foresters.

Thank you, we have adjusted all referenced cases throughout the manuscript to “aged”.

Introduction General: I suggest adding a paragraph (or even just a couple sentences somewhere) to highlight the types and reported effects of commonly-applied fuel reduction/fire resilience treatments that have broad support in other regions/ecosystems as a way to (1) provide context and support for the “fire-aware silvicultural treatments” you included in your simulations, and (2) highlight the discrepancy between common conceptions (misconceptions?) about the potential efficacy of fuel treatments in moist, westside forests of the PNW and a literature base that suggests some treatments (e.g., broadcast burning plus thinning) pretty consistently reduce fire severity across a wide array of biophysical environments outside of the most extreme fire weather conditions.

Thank you, we’ve added the suggested paragraph into the introduction (now lines 134-149).

L75-94: It might be helpful to provide a figure (e.g., a pie chart) that shows the land ownership breakdowns described here.

Thanks for the suggestion, we have integrated a pie chart inset graph into Figure 1 that shows the land ownership areal breakdowns.

L79-82: Is active forest management on NFS lands rare, or just constrained to a small percentage of the landbase (i.e, primarily in plantations that fall within NWFP matrix and AMA land use allocations), and a limited set of activities that have relatively broad social license (i.e., thinning, pile burning, occasion broadcast burning, and planting after wildfires)?

Thank you for the comment, we have edited the sentence here (now lines 96-101) to provide some quantitative evidence regarding active management on NFS lands and acknowledge that some timber resource management does occur: “*Forest Inventory and Analysis (FIA) data indicate that active management (thinning, clearcutting, and other silvicultural activities excluding incidental cutting) has occurred in recent history (past ~30 years) on less than 24% of unreserved westside NFS forestland. While active management for timber resources does occur on a subset of NFS lands, hazard tree removal (e.g., along roadways, powerlines, campgrounds, and recreational areas) is perhaps the most commonly observed management action of the agency at a landscape scale”.*

L93-94: It is misleading to imply that the term “regeneration harvest” equates to clearcutting. Clearcutting is the regeneration harvest method that is overwhelmingly used on industrial forestlands in the PNW, but there are many other regeneration harvest methods (e.g., seed tree cuts, variations on shelterwoods, retention harvests, and several variants on selection cutting methods). Consider something along the lines of “plantations of Douglas-fir… managed using clearcut harvests on approximately 35-60 year rotations” to avoid perpetuating the misconception that a regeneration harvest always means a clearcut.

Great point, we have edited the text here as suggested.

L95-96: The sentiment of this statement is accurate (i.e., that fuel management techniques have not been widely utilized in moist, westside forests of the PNW), with one exception: slash burning is commonly practiced after clearcuts and sometimes practiced after thinning within this region. While this certainly doesn’t represent a comprehensive fuel management program, and may be done as much to facilitate planting as to reduce fuel loading, pile burning (and the broadcast burning of slash that was common in the region prior to the 1980s) is a common practice with a supporting research based that goes back to at least the 1950’s (e.g., Morris, M.G. 1958. Influence of slash burning on regeneration, other plant cover, and fire hazard in the Douglas-fir region. Research Paper No. 29. USDA Forest Service Forest and Range Experiment Station. Portland, OR). I suggest simply acknowledging that there has been relatively common use of slash removal as a site preparation technique following clearcut harvests in the region (perhaps referencing a couple of associated studies), while fuel treatments in existing stands have been rare.

Thank you for the comment, we have added the following sentence after this line (now lines 112-114) to make this point more explicit: *“Pile burning of slash commonly follows clearcut activity to facilitate planting and reduce fuel loading elevated by harvest operations”.*

L269-272: I suggest a minor wording revision to clarify that the silvicultural practices described were the examples of uneven-aged and even-aged silvicultural practices that you chose to simulate. For instance, “Treatments were associated with representative examples of either even- or uneven-age management styles, where the former was simulated using clearcutting (both with and without commercial thinning?) on varied rotations and the latter was simulated using repeated individual tree selection harvests, with trees thinned evenly across diameter classes in each entry, to maintain recruitment of new, younger growing stock over time.” Please note that my use of the terminology “individual tree selection harvests” instead of thinning would be appropriate if your repeated entries were associated with regeneration establishment objectives. If not, then “repeated commercial thinning” is fine, but isn’t actually representative of uneven-aged management. It would just be repeated thinning.

Thanks for the suggestion. We have revised the sentence accordingly.

Methods and Connections to Results (General): Several of the primary results describe stand-level outcomes, while others describe landscape-level outcomes that are reflective of proportions of stands that meet some criteria, or (for NPV) presumably the cumulative NPV across all stands. This is fine, but I don’t see a clear rationale for why the results that represent stand-scale statistics (i.e., means of stand-level values) haven’t been compared with an appropriate statistical model. The authors should provide either some rationale for this choice, or adopt an appropriate statistical modelling framework to support inference from comparisons of stand-scale means. A GLM framework (or perhaps LMM with random effects for ecoregion or some other broad quantifier of spatial and biophysical proximity) seems perfectly acceptable here.

Thank you for the comment. First, we want to clarify that it sounds like what the reviewer is asking for are tests for statistically significant differences between groups. One does not use statistical predictive models for this purpose as suggested (e.g., LMM or GLM) – the purpose of using those models would be to assess the direction and magnitude of the statistical relationship between a set of predictive variables and a response variable. That alternative type of analysis is explicitly not the focus of our study – we are only summarizing outcomes, not trying to understand what specifically is driving outcomes and whether those variables exhibit a statistically significant relationship. Given that correction, our rationale and choice to not comprehensively test and report on statistical differences between groups across all outcome metrics and in all figures was as follows:

1) Our purpose was to understand treatment effectiveness, economics, and trade-offs against climate benefits achieved via carbon sequestration and storage; it was not to accept/reject null hypotheses concerning differences between age/structure class and land ownership groups. This is well-represented in how we define our research questions. Further, statistical significance values will depend on class definitions (e.g., FIA recorded stand-age and forest structure class, both of which have complex definitional nuances and imprecision/arbitrariness in application) and sample size (which varies by nearly two orders of magnitude across groups). We worry that lack of statistical difference between any two widely separated and policy-relevant groups may be solely due to small sample size, while existence of statistical difference may be solely due to a large sample size that makes even tiny differences statistically significant, but not necessarily of great policy relevance.

2) Adding symbolization of the results associated with tests for statistical differences, across groups in all the outcome figures, adds an additional level of complexity in graphs that are already quite complex. We believe that comprehensively symbolizing those test results across figures would critically reduce their interpretability; we tried including this kind of symbolization earlier in the study and abandoned it after receiving negative feedback from study collaborators and stakeholders regarding figure interpretability.

As a compromise and as recommended by Reviewer 2, we have incorporated tests for statistical differences between groups (within initial age/structure classes between management scenarios; Wilcoxon Rank Sum tests appropriate for non-normal response distributions and unequal sample sizes between groups) into the outcome metric tradeoffs analysis presented in Table 3 (was Table 5). Potential differences between groups are symbolized by superscript letters attached to the area-weighted medians.

L655-657: The claim here that uneven-aged treatments produced less vertical canopy continuity is puzzling. In general, I would expect even-aged stand structures to have more uniform, single-strata canopies, while the multi-cohort structures that result from uneven-aged management, and the associated variability in tree diameters, heights, and crown positions should tend to produce more vertical connectivity. Additionally, I would expect the initiation of repeated thinning to delay crown recession, which would also tend to promote connectivity between younger understory to midstory tree cohorts and an older cohort of larger trees. At least from a diameter distribution standpoint, multiple studies suggest uneven-aged silviculture results in more varied tree sizes than even-aged management (e.g., Goodburn and Lorimer 1999 – Forest Ecology and Management, Janowiak et al. 2008 – Forest Science), and this is also implied by diameter distributions reported in several uneven-aged management studies in Douglas-fir forests of the PNW (Miller and Emmingham 2001, Ralston et al. 2004, Curtis 2010), one of which was co-authored by a co-author on this manuscript. In general, these more varied/less normally-distributed diameter distributions are associated with more varied tree heights and crown positions. Thus, I wouldn’t expect reduced vertical canopy continuity in stands managed with uneven-aged silvicultural approaches. Perhaps the authors could provide height distribution data to support this claim?

Alternatively, increased fire resistance across a population of stands managed using uneven-aged management, could be explained by other mechanisms. For instance, in uneven-aged systems all stands tend to have a significant proportion of their SDI (or volume since that was the metric used for characterizing fire resistance in this study) in larger, more fire resistant trees, while across a population of stands managed using even-aged techniques, a significant proportion of stands will always be younger stands with most of their SDI and volume in small-diameter trees with limited fire resistance. This provides what may, perhaps, be a more intuitive rationale for uneven-aged management to provide increased fire resistance at the landscape scale, and is consistent with later points in the discussion about the lack of fire resistance in young, even-aged plantations.

We regret the errant explanation – thank you for catching that. We have replaced it.

L728: Consider revising to read: “Under fire-focused management, carbon sequestration decreases slightly…

Adjusted as suggested.

**Reviewer #2**

**Summary:**

In this manuscript, Busby and Fried seek to characterize potential tradeoffs between fire resistance, carbon, and economic goals with regard to forest management strategies in the western Cascades, using a simulation modeling approach. The authors compare business-as-usual and fire-aware management approaches across a variety of land ownership and forest structure scenarios. The authors note potential considerations for future management based on these results.

I think that this study will be of interest to both researcher and manager readership of *PLOS ONE*, and is appropriate for this journal’s aims and scope. Overall I think this study is timely and well-designed, but would benefit from additional edits for clarity and conciseness before publication. I have outlined my recommendations below and look forward to seeing the next version of the manuscript.

**Main comments (essential revisions):**

1. At 26 pages, 15 figures, and 5 tables, the paper is quite long and in places feels unfocused. I can appreciate the challenge with concisely reporting findings for such a large, multidimensional model. That said, I suggest editing with an eye toward trimming the paper back to its main story. I have made some specific suggestions below, but consider limiting figures to one per results section (i.e., one carbon figure, one fire resistance figure, one economic figure), paneling or summarizing results as necessary. I would also consider actually adding a figure to the tradeoffs section (either in addition to or in place of the table). To me this is the “star” of the paper and it would be great to have a visual here that is digestible at a glance.

   Thank you for the valuable feedback; we have worked through the manuscript, guided by review comments, to revise/refine/focus the writing as possible, while acknowledging the challenge of reporting on a very complex / multidimensional modeling study in a concise manner. We have focused on moving non-essential tables, figures, and text to the supplementary materials as possible. This included 2 tables, 6 figures, and several paragraphs of text from the Forest inventory data and Silvicultural treatments sections in the Materials and Methods, into a new S1 Appendix.

Adding further paneling dimensions to aggregate figures is likely to render figures overly complex and harder to interpret, which influenced our decision to instead move 6 non-essential figures (stand and landscape scale carbon storage and merchantable / non-merchantable harvest volume) to the supplementary materials. We originally attempted to symbolize the tradeoff outcomes via a figure graphic, but the multidimensionality of the data and comparisons did not support an easily interpretable figure – hence we pivoted to summarizing those outcomes in a tabular format (Table 5, now Table 3). Given the existing number of figures is very high, we would prefer not to further inflate that number with an additional figure.

2. The First Order Fire Effects Model is a major part of this study. However, as far as I can tell the first mention of this model is in Table 3, with no information on how this model was used or parameterized in the methods. I feel that I cannot adequately evaluate this portion of the study without more information.

   Thanks for pointing out that deficiency. We have now introduced the fire resistance variable, its derivation and others used in the optimization, in the Simulation outputs metrics section.
3. It is not clear to me whether each individual model scenario was run multiple times or just once. If multiple times, how much variability in outcomes is there per scenario? If just once, how did you account for stochastic effects in the model?

   Sorry that this wasn’t clear – we used a deterministic modeling approach where a single FVS simulation (without a random seed; same results if simulation was re-run) was used to represent each scenario. This has been clarified briefly in the methods (now lines 266-267). Practical modeling limitations (data size, modeling time, and post-processing workflow through the BioSum software) prevented us from pursuing a stochastic modeling approach.
4. Although results are thoroughly described, the paper is lacking in statistical testing in general. It would be especially helpful to see some additional stats in the tradeoffs section of the results. Are outcomes significantly different by initial forest structure and/or management scenario?

   As suggested, we have incorporated tests for statistical differences (via pairwise Wilcoxon Rank Sum tests) between groups (within initial age/structure class between management scenarios, for each outcome variable) in Table 5 (now Table 3) – these are symbolized as superscript letters attached to the area-weighted median estimate.

**Minor comments (desirable revisions):**

1. The simulation period (40 years) does not include the full range of a single rotation period (35-60 years), which I feel is a crucial caveat to interpreting the results. While this is addressed in Lines 738-750 of the discussion, I would like to see a bit more discussion of the effects of simulation length on outcomes other than carbon alone (i.e., potential differences between short-term and long-term tradeoffs, especially in the context of repeat burns). Although this is touched on in the discussion, it may also be worth briefly noting in the methods (Line 295) why 40 years was chosen.

   Thanks for this useful suggestion. We now explain the choice of simulation period in the methods and discuss the interaction of simulation length with initial stand age at the beginning of the results where the treatment assignment patterns are described. This paper does not directly address reburns in that while we do simulate burns of different severities at different times, and combine those multiple fire and non-fire simulations into an expected value outcome, we do not simulate multiple, sequential burns in the same stand during the 4-decade simulation.
2. There are several sentences in the methods and results section that are difficult to parse – I have outlined a few in the following section. Consider breaking up clauses into sentences and reducing parentheses use where possible.

   Thank you for the feedback, which we’ve used to rework nearly all paragraphs in the methods to enhance clarity, focus and brevity.
3. There are a great many acronyms in this paper. Although they make sense in cases where they are defined appropriately and used frequently (like BAU, FF, and FFCA), others (like LCA and FFE) are only used a handful of times and should be written out.

   Thank you, we have attempted to remove infrequently used acronyms throughout the manuscript.
4. In general I would like to see more quantitative reporting in the results, especially if the number of figures is reduced. Statements such as “exceptionally low” (Line 490), “modest improvements” (Line 511), “lower tree survival” (Line 515), “more modest” (Line 535), etc. should be replaced with the corresponding value or statistic where feasible.

   Noted, we have attempted to provide more quantitative estimates (e.g., 20-150% increase in median value; ~30% decrease in area) in the text across the Results section.
5. It was difficult for me to parse that the FF and FFCA scenarios fall under the FA category presented in Table 1. I would recommend clarifying this in 397-399.

   Thank you, this has been clarified in the text.

Specific text comments

- Lines 40-51: This paragraph lacks citations; I would recommend at least adding an appropriate citation for the sentence beginning with “Westside forests…” (lines 44-47).

  Thank you, an appropriate citation has been added here (now line 47).
- Lines 107-109: Not an edit – just noting that I really like this sentence.

  Thank you!
- Lines 144-169: Some of this information is already stated in the introduction; I recommend condensing where possible.

  Good point. While there is some overlap with the introduction text, we believe this paragraph adds important context for interpreting the results with regards to forest composition and productivity gradients, and how those interact with land ownership/management, that is not well explained in the introduction section.
- Lines 213-215: I am not sure what is meant by “used stand inventory conditions to select and enter an appropriate code”.

  This has been further clarified in the text: *“When the PV_CODE field was null or contained a code regarded as invalid for FVS, we used stand inventory conditions* ***(species composition, location, and site productivity) to crosswalk each stand to an appropriately matched code.****”* This text was additionally moved to the S1 Appendix to reduce manuscript length.
- Lines 220-247: This paragraph is very long – consider breaking up at line 231 (“Interviews with…”)

  Agreed, paragraph has been split here.
- Lines 251-254: I am not sure what is meant by this sentence.

  Sentence has been revised for clarity.
- Line 300: Clarify why these wind speeds were selected.

  Additional context (adjusted wind speeds better represent observed regional fire weather conditions) has been added in this sentence and the following to provide clearer logic supporting the decision. We’ve redefined the “moderate” fire weather scenario as “mild-moderate”, to better represent the actual fire weather parameters used.
- Lines 320-328: Consider moving these lines to the “Wildfire Activity and Prevalence” section.

  Good suggestion, we have moved and integrated these lines into the “Wildfire Activity and Prevalence section.
- Lines 386: Why was Year 41 unevenly weighted?

  Weights are designed to evenly split the decades between a simulation result from shortly after treatment to shortly before the following treatment. Because the last treatment in BioSum occurs at year 32 and there is no post-treatment simulation result available owing to the architecture of the model, we rely on the final simulation result at the end of the 4^th^ decade to represent that decade. We have updated the text to clarify.
- Line 475: Consider changing 18% unmanaged to 82% managed, for consistency and easier comparison with the preceding paragraphs.

  Thanks, this has been adjusted as suggested.
- Line 550: Should this be FF instead of FA?

  Yes, this has been corrected.
- Line 578 and elsewhere: Consider changing “stand level” and “landscape level” to “stand scale” and “landscape scale” for clarity.

  Thanks for the suggestion, we’ve changed references of stand and landscape level to scale throughout the manuscript.
- Lines 635-650: Lots of background information here before a reference to your findings – consider removing this paragraph altogether or moving to the introduction.

  Great suggestion, we have moved this paragraph into the Introduction section (now lines 66-79).

Figures & tables

- Figure 1 – the color schemes here are a bit confusing, as the colors for “State” and “Mature Even”, “Private” and “Young Uneven”, and “NFS” and “Supermature Even” are very similar. It is easy for readers to look at the wrong legend.

  Thanks for pointing this out. In Figure 1, we’ve re-symbolized the color scheme for the area by age/structure class summary data (now a pie chart) to better differentiate from the ownership color scheme, as well as added a new pie chart that summarizes area by ownership, including Corporate and Non-Corporate subclasses of Private ownership. We do not make the differentiation between these two subclasses when symbolizing the fuzzed location of the FIA plots because this spatial information is confidential, even when fuzzed.
- As previously noted, consider further paneling violin plots per result topic or moving some of these figures to the supplement.

Noted, we have moved 6 non-essential figures to the supplementary materials.

- Table 1 – define what “trigger” means in the model context either in the table caption or somewhere in the text.

  Great suggestion, we have added definitions for “trigger” and “target” at the bottom of Table 1.
- Table 4 could also be moved to the supplement as it is not strictly necessary for interpreting the modeling process, in my opinion.

  Thanks for the suggestion, we have moved Table 4 to the supplementary material (now S3 Table).
