# Supplementary material for: Prospects for silvicultural enhancement of fire resistance in mesic westside forests of the Pacific Northwest
Source: PLoS One. 2025 Sep 8;20(9):e0332158. doi: 10.1371/journal.pone.0332158 (PMC12416676; doi:10.1371/journal.pone.0332158)
Supplement: Attachment — Submitted filename: Response_to_Reviewers_auresp_2.docx [file pone.0332158.s017.docx]

**See author responses to remaining reviewer comments below in blue text.**

Reviewer #1: I commend the authors for making a number of significant changes to the manuscript in response to the reviewers' comments on the initial submission. The addition of more nuanced descriptions and discussion of common silvicultural treatment regimes and implications of the paper's results across different ownerships in the introduction and discussion, the addition of relevant statistical analyses to their tradeoffs assessment, and the overall streamlining and re-focusing of the discussion have greatly improved the manuscript. This revised manuscript offers compelling insights and consideration of what “fire-aware” management might look like in moist, westside PNW forests, and what the implications are for a variety of resource values. I have only a few minor suggested to consider before the eventual publication of the manuscript.

L426-435: This interpretation and explanation of the results would be more appropriate for the discussion.

Good point, we have removed this short paragraph from the results section.

L586-593: I applaud the author’s addition of appropriate statistical tests for their tradeoff analyses, but some version of this explanation of how they treated and analyzed the data should be incorporated into the methods section.

Great point, we have expanded upon and moved this methodological explanatory text into an “Evaluating tradeoffs” section at the end of the methods section.

Reviewer #2: (No Response)

Reviewer #3: I found that the authors have addressed the comments very well and substantially revised the manuscript, and it is acceptable for publication. However, I have some concerns
Some Acronyms, such as FF, FFCA, Rx, and others, have been repeatedly spelled out throughout the paragraph. Is this a common standard or something else? Please fix this issue.

Good point. Our intention was to reintroduce acronyms as appropriate in each new section (to improve reader comprehension, given there are a lot of acronyms used), but you’re right that the usage is somewhat inconsistent throughout. For consistency, we have revised the manuscript so that the first use of an acronym is spelled out in each major section (i.e., Introduction, Methods, Results, Discussion, Conclusions) and all following cases within a section are stated only in acronym form.

I suggest using the full y-axis range while showing the range of distribution, for example, Fig. 7, where the upper and lower quantile range is cut off in some violin, due to the constrained y-axis range. Check the similar issue with Fig. 9, and S3, S5, S7

As suggested, we have adjusted all the relevant stand-scale violin plot figures (7, 9, S3, S5, S7) so that the y-axis is no longer constrained.

Fig 8- provide a negative y-axis value for the 6th right panel (Washington); as well Fig S4.

Given the y-axis range and tick spacing across panels are flexible in figures 8 and S4 (no common scale across panels), we cannot add (specify) an additional negative y-axis tick value in the plotting package (ggplot2) used to generate these graphs. Given the y-axis tick spacing is systematic, however, readers can reference the horizontal lines in the graph to interpret negative values (e.g., in the panel referenced in Figure 8, observed negative values range out to half the systematic y-axis tick spacing or to a value of -0.5).

**Additional Author Note:** Upon review we found that the trees per hectare (TPH) estimates stated throughout Table 1 (silvicultural prescriptions) were incorrect due to a conversion factor mistake. These estimates were originally obtained in trees per acre (TPA) and were mistakenly divided by the acres to hectares conversion factor (~2.471) rather than multiplied. All TPH estimates have been properly converted and revised in Table 1.
